# Evaluation of Crestal Bone Loss in Implant-Retained Mandibular Overdentures With Locator Attachments: Immediate VS. Delayed Loading Protocols

**DOI:** 10.7759/cureus.97400

**Published:** 2025-11-21

**Authors:** Abdel-Fattah E Mohamed, Ehab Atito, Esmail Ahmed Abdel-Gawwad, Mostafa I Fayad, Wesam E Badr, Adel F Abd El Hakim, Mohamed A Helal

**Affiliations:** 1 Dentist, Egyptian Ministry of Health, Cairo, EGY; 2 Removable Prosthodontics, Faculty of Dental Medicine, Al-Azhar University, Cairo, EGY; 3 Substitutive Dental Science, College of Dentistry, Taibah University, Medinah, SAU

**Keywords:** crestal bone loss, delayed loading, immediate loading, implant overdenture, locator attachment

## Abstract

Objective

The research sought to assess the effects of loading dental implants immediately compared to using a delayed loading approach on peri-implant crestal bone loss in patients wearing implant-retained lower dentures with locator attachments (after six months and 12 months).

Subjects and methods

A group of twenty fully edentulous patients was randomly chosen and divided equally between two groups (n=10): Group I, delayed loading (DLO) received a delayed-loaded implant-retained mandibular overdenture with a locator attachment, while Group II, immediate loading (ILO) received an immediately loaded implant-retained lower overdenture with a locator system. The loss of bone around the implant at the crest level was evaluated utilizing intra-oral digital radiographs obtained through the long cone paralleling technique at the time of implant loading, six months, and 12 months after loading. The statistical analysis of the results was performed through the Mann-Whitney U-test and Wilcoxon's signed-rank tests, with significance set at P≤0.05.

Results

The findings showed no statistically significant differences in bone loss around the implant crest between immediate and delayed loading approaches (P≤0.05).

Conclusion

Within the limitations of the present study, it is concluded that the peri-implant crestal bone loss associated with the immediate loading protocol for implant-retained mandibular overdentures utilizing locator attachments demonstrated no statistically significant difference when compared to the delayed loading protocol at the one-year follow-up period.

## Introduction

Managing mandibular dentures is challenging, necessitating extensive research into different impression techniques and occlusal schemes. Current perspectives suggest that conventional complete dentures may be inadequate, advocating for implant-retained overdentures with two anterior implants as the standard care for edentulous mandibles [1, 2].

A dental implant is an alloplastic prosthetic that supports and retains a dental prosthesis that is either fixed or removable. It is placed into the oral tissues beneath the mucosal or periosteal layer and on or into the bone [3].

Brånemark initially advised a healing period of four to six months before lower implants are loaded [4]. Clinical guidelines and general recommendations for implant loading regimens have been released by Gallucci et al. [5]. An implant-supported restoration is said to be immediately loaded if placed within 48 hours of the implant being placed, ensuring the opposing dentition and the occlusal surface are in functional contact. The locator attachment’s low-profile design is popular among patients with limited inter-arch space, as it minimizes denture base deformation and fracture [6]. It exhibits a high retention value compared to ball attachments, owing to its dual retention mode, which increases the surface area of retentive contact [7].

The loss of bones around dental implants at the crest level is thought to be caused by multiple factors and may happen either soon after placement or later during the implant's lifespan. Early crestal bone loss, defined as occurring within the first year post-implant placement, is attributed to bone remodeling following surgical and prosthetic treatments, as well as the early loading issues faced with the implant and the related prosthetic [8].

The loss of bone at the crest level surrounding dental implants plays a crucial role in determining the long-term effectiveness of implant treatment. It has been reported that a bone loss of 1.5 mm within the first year post-loading can be considered successful if the ensuing yearly loss of bone is less than 0.2 mm. The observation of minimal crestal bone loss prompted the suggestion of favorable factors for the success of implant therapy. These criteria, which include ≤1-2 mm of crestal bone loss within the first year following placement and ≤0.2 mm average of crestal bone loss throughout the following years, were promptly embraced by academics and scholars throughout the world. Initially, implant placement in individuals who were partially edentulous raised concerns, the fact that these criteria were based on clinical results of implant therapy in edentulous patients or subjects. However, there was still concern that implants placed in partially edentulous patients, with their dental reservoirs of periodontopathogens, would not be able to replicate the excellent crestal bone response observed in edentulous patients [8, 9].

It has been demonstrated that implant therapy following Brånemark’s approach exhibits the same exceptional resistance to crestal bone loss in participants who are partly edentulous as those who are edentulous. However, in the comparatively rare instances of crestal bone loss, bacterial cultures, including conventional periodontal pathogens and contemporaneous peri-implant mucosal inflammation, led to the erroneous belief that peri-implant bone loss and periodontitis are essentially the same [10].

The choice of loading protocol, immediate versus delayed, has been a subject of considerable debate among clinicians and researchers. Immediate loading protocols provide better patient satisfaction and shorter treatment times, but concerns remain regarding their impact on peri-implant bone stability. Conversely, delayed loading protocols traditionally provide a more favorable environment for osseointegration, possibly reducing the chances of initial implant complications and loss of bone at the crest level [11].

This study highlights the use of the locator abutment (Dentium Co. Ltd., Seoul, South Korea) attachment system due to its self-aligning design and proven clinical reliability. By evaluating crestal bone changes at six and 12 months, the study provides insight into the short-term and early mid-term osseous remodeling phases. The primary objective of this research was to assess and compare the crestal bone loss around implants supporting mandibular overdentures using locator attachments under immediate versus delayed loading protocols. The null hypothesis proposed that there would be no significant difference in crestal bone loss around implants amongst immediate and delayed loading procedures when using locator-retained lower overdentures.

## Materials and methods

Twenty fully edentulous patients were randomly selected from the outpatient clinic of the Prosthodontics Department at Al-Azhar University's Faculty of Dental Medicine (Boys, Cairo) in Egypt to participate in this study. A thorough medical history, clinical examination, and radiographic assessment were performed for the selected patients to identify conditions that might affect their suitability for surgery and implant survival.

Before enrollment in the study, all patients provided informed consent after being thoroughly informed about the study methodology. The Al-Azhar University Faculty of Dental Medicine Research Ethics Committee approved the study under the reference number EC Ref No. 895/185.

Based on a previous study, a sample of twenty completely edentulous patients was selected [12]. The G*Power statistical power analysis software (version 3.1.9.4, Heinrich-Heine-Universität Düsseldorf, Germany) was used to do a power analysis. For the two-sided hypothesis test, a total sample size of n=20, split into two groups of 10 each, was considered enough to detect a big effect size (f=0.92), with an actual power (1−β1 error) of 0.8 (80%) and a significance level (α error) of 0.05 (5%).

All patients had to meet the following inclusion criteria: patients were included if they were completely edentulous in the mandible, were medically controlled American Society of Anesthesiologists (ASA) I-II patients, were free of any medical conditions that may affect the bone or risk the implant placement surgery, and had healthy mucosa. Furthermore, participants were required to have adequate bone volume to accommodate implants of at least 3.6 mm diameter and demonstrate both willingness and capability to comply with all study requirements. The exclusion criteria for the patients included: uncontrolled systemic disease that might compromise implant surgery, a history of chemotherapy or radiotherapy to the head and neck region, and heavy smokers.

The chosen participants were randomly assigned into two equal groups: Group I, delayed loading [(DLO) (n=10)], which received delayed loading mandibular complete overdentures, and Group II, immediate loading [(ILO) (n=10)], which received immediate loading mandibular complete overdentures. The allocation of participants to the treatment groups was performed using a rigorous randomization process to minimize selection bias. A computer-generated random sequence was utilized to create the allocation list. Subsequently, this sequence was concealed using sequentially numbered, opaque, sealed envelopes. Once a participant met the inclusion criteria and provided informed consent, the next available sealed envelope was opened to reveal their assigned group (DLO or ILO), thereby ensuring allocation concealment before intervention.

Before implant surgery, each patient received a newly fabricated set of complete dentures for both the upper and lower arches with a bilateral balanced occlusion. After denture placement, patients received care instructions and were advised to maintain continuous denture wear until adaptation was achieved.

Before the surgical intervention, all patients received antibiotic prophylaxis. The choice of antibiotic was based on the presence of a penicillin allergy: either 2 grams of amoxicillin or 600 milligrams of clindamycin was administered. Patients were directed to rinse their mouths with 0.25 mg/ml chlorhexidine. The circumoral skin was cleansed with 70% alcohol and 10% povidone-iodine. Local anesthesia was achieved using a bilateral mental nerve block and lingual infiltration with 2% articaine hydrochloride and 1:100,000 epinephrine (Inibsa, Inibsa Dental, S.L.U., Barcelona, Spain)

A transparent acrylic replica of the mandibular denture was used to create the surgical template, which was meticulously adjusted in the canine region to facilitate initial cortical bone penetration. This was achieved by using a round bur through the aperture in the surgical template, which precisely corresponded to the intended implant placement site.

A flap of full thickness was raised to the mucogingival junction to minimize post-operative swelling and facilitate easier access to the implant site using a periosteal elevator. The initial osteotomy was created with a pilot drill, using a copious amount of saline solution until the desired implant length was reached. Two osteotomies were prepared bilaterally at the canine region. The osteotomies were completed following the sequence of drills in the selected kit. Two dental implant fixtures from the Superline Implant System (Dentium Co., Ltd., Seoul, South Korea) were placed at the osteotomy sites, each with a length of 12.0 mm and a diameter of 3.6 mm. All implants placed in both DLO and ILO groups achieved primary stability with insertion torque ≥35 N⋅cm [13]. All implants were placed by a single experienced surgeon, strictly adhering to the established surgical protocol.

DLO group

**Figure 1:**
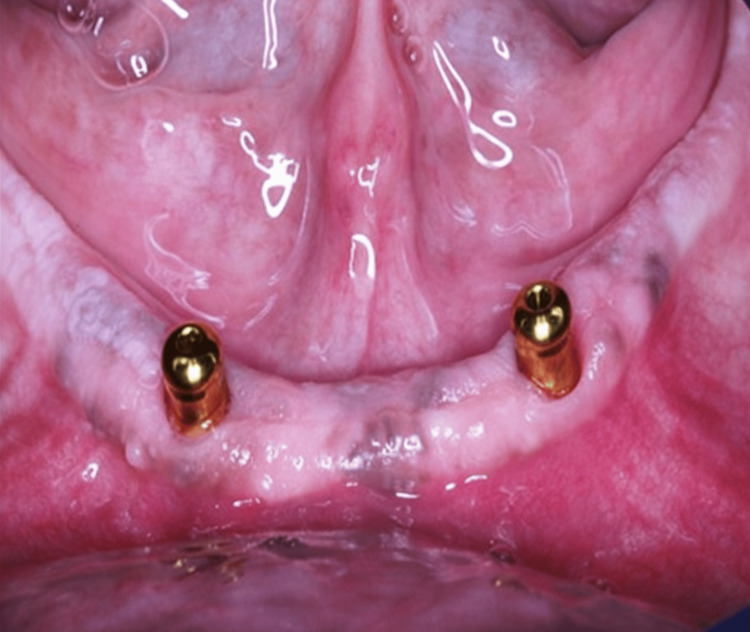
Locator Abutments Connected to the Implant after Three Months

The cover screws were fastened to the implants, and the flap was sutured. Three months post-surgery, the healing abutment was placed and subsequently replaced with the locator abutment two weeks later. The locator abutment was tightened with a torque of 25-30 N.cm according to the manufacturer's instructions (Figure 1).

Metal caps were fitted onto each locator abutment, and their positions were marked on the denture using a colored indicator paste. When the lower denture was placed in the mouth, it created impressions showing the exact location of the caps.

The mandibular complete denture required modification to create space on the impression surface to accommodate the metal cap, which was achieved using a large round bur. Cold-curing acrylic resin (Acrostone, Egypt) was used to attach the metal cap to the denture-fitting surface. The patient was urged to gently occlude in a centric position once the denture was put in their mouth. After the self-curing acrylic resin had set, the overdenture with the metal cap was removed and examined. Then, any excess material was trimmed away before being polished to a smooth finish.

ILO group

In Group II (ILO), the locator abutment was immediately tightened to the implants after surgery with a 25-30 N.cm torque. The flap surrounding the abutment was then enclosed and sutured.

A metallic housing was positioned atop the locator abutment, and its position was transferred to the denture using a marking paste applied to the cap. The mandibular denture was then seated over the ridge, marking the corresponding metal cap to the denture’s tissue surface.

To accommodate the fitting surface for the metal cap, space was created in the mandibular complete denture using a large, round bur. Using self-cure acrylic resin, the metal cap was directly connected to the denture fitting surface. After placing the denture, the patient was instructed to close in centric occlusion. Following the complete setting of the self-curing resin, the overdenture was removed, inspected, and polished until smooth.

All patients were provided with antibiotics (125 mg of clavulanic acid and 875 mg of amoxicillin) to be taken twice daily for seven days following surgery. Every participant was instructed to use mouthwash containing 0.2% chlorhexidine three times a day. For 10 days, a soft, cold diet was advised.

Crestal bone loss evaluation

Radiographic measurements were performed by one calibrated examiner, blinded to group allocation, using a standardized long-cone paralleling technique. A sensor holder with a positioning system (DR Comfy, Engineering Solutions Ltd, Egypt) was used to maintain consistent film-to-implant and cone-to-implant distances during subsequent exposures. The film sensor (Handy-HDR-500, Shanghai 201906, P.R., China) was exposed, then scanned and stored.

Measuring mandibular crestal bone loss around the implant

Digital periapical radiographs were captured at three intervals: immediately after implant loading (baseline) and at six and twelve months following the loading. The evaluation radiographs were obtained using the parallel cone angle technique, adhering to a standardized protocol [14].

Using the HandyDentist V3.31.02 software interface, the distance from the crest of the ridge to the apical end of the implant body was measured perpendicularly on both the distal and mesial sides of each implant. Additionally, the long axis of the implant was used to measure its length on digital radiographs. By comparing the length of the implant as seen in digital intraoral periapical radiographs with its actual physical length and using the crestal bone level readings on both sides of the implant, the measurements were normalized using the formula provided by Patil and Nimbalkar-Patil [15].

Bone level at baseline = [(Crest bone level reading at baseline x Physical length of implant body)/length of the implant reading at baseline] [15, 16].

**Figure 2:**
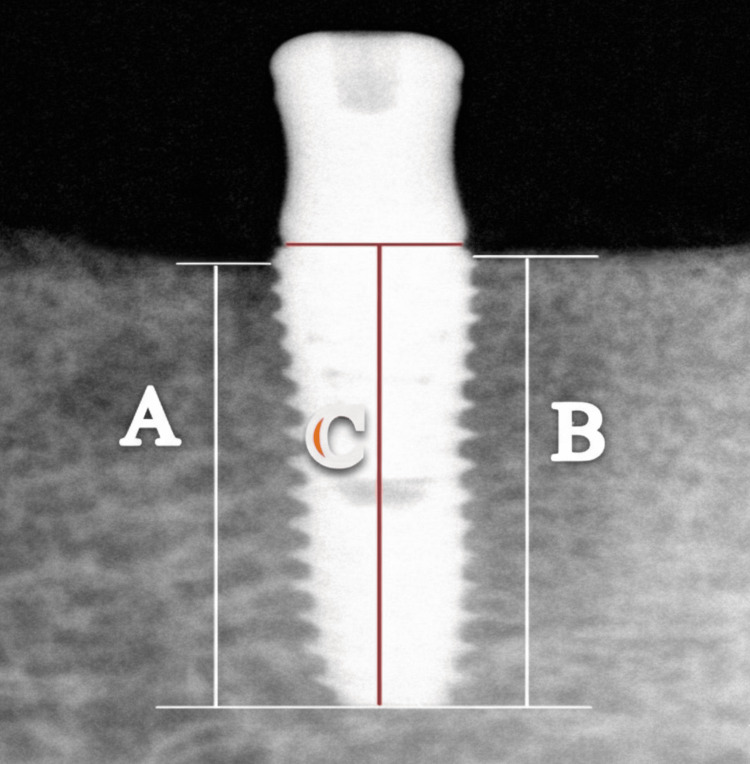
Crestal Bone Height Measurement A and B represent the distance from the ridge crest to the apical end of the implant body, measured at the mesial and distal aspects, respectively. C represents the implant body height.

Digital intraoral periapical radiographs were used to take three measures for each implant at baseline (when the implant was loaded) and recall follow-up visits six and twelve months later. Crestal bone loss was defined as the vertical distance (in millimeters) between the implant platform and the first bone-to-implant contact measured on standardized radiographs. The extent of crestal bone changes was measured in millimeters (mm) around the implants, and was determined by comparing the normalized baseline measurements with those taken at six months. Thus, bone-level changes were assessed for each implant at both six months and twelve months. The mean of the two values from both the mesial and distal sides was used for statistical analysis [16] (Figure 2).

SPSS version 20.0 (IBM Corp., Armonk, USA) was used to conduct the statistical analysis. The Kolmogorov-Smirnov test was used to assess the normality of the data. Due to the non-normal distribution of the data, non-parametric statistical tests were employed for group comparisons. The Mann-Whitney U-test was used for comparisons between groups, while Wilcoxon's signed-rank test was utilized for paired observations within each group. Statistical significance was established at (P≤0.05).

## Results

Crestal bone height evaluation

Table 1 displays the mean crestal bone height measurements for both groups across different periods. Analysis revealed significant progressive bone loss, with notably decreased bone height values at twelve months compared to initial implant loading. The results showed significant variations in crestal bone measurements between six and twelve months (P=0.000*).

**Table 1:** Comparison of crestal Bone Height Changes for Right and Left Implants at Baseline, Six Months, and Twelve Months Post-loading. DLO: delayed load; ILO: immediate load; analysis of variance (ANOVA) test was used for within-group comparisons. * Denotes significance at P<0.05.

	Group	Baseline		At 6 months		At 12 months		
		Mean	Std. Deviation	Mean	Std. Deviation	Mean	Std. Deviation	P-Value
Right implant	DLO	11.49	0.57182	11.28	0.57992	10.75	0.54384	<0.001*
	ILO	12.08	0.20525	11.83	0.2323	11.31	0.22476	<0.001*
Left implant	DLO	11.51	0.54832	11.3	0.56334	10.77	0.52153	<0.001*
	ILO	12.03	0.46524	11.78	0.48717	11.26	0.42573	<0.001*

Change of crestal bone height evaluation between different intervals

The change in crestal bone height evaluation between different intervals of each group is listed in Table 2.

Regarding the right implant's crestal bone height, changes revealed no statistically significant variations across the groups during both time intervals: from implant loading to six months (P=0.196) and from six to 12 months (P=0.839).

Regarding the left implant, comparative analysis showed no statistically significant variations in mean crestal bone loss across groups during both periods: from initial loading to six months (P=0.242) and from six to 12 months (P=0.927).

**Table 2:** Comparison of Crestal Bone Height Changes Between Groups for Both Right and Left Implants DLO: delayed load; ILO: immediate load; Independent-samples *t*-tests were used for comparison between groups. Significance is set at P<0.05; ns=not significant.

		Group	Mean	Std. Deviation	Std. Error Mean	P-Value
Right implant	From loading to 6 months	DLO	0.2115	0.05667	0.01792	0.196 ns
		ILO	0.25	0.07071	0.02236	
	From 6 months to 12 months	DLO	0.5305	0.11485	0.03632	0.839 ns
		ILO	0.52	0.11353	0.0359	
Left implant	From loading to 6 months	DLO	0.215	0.05798	0.01833	0.242 ns
		ILO	0.25	0.07071	0.02236	
	From 6 months to 12 months	DLO	0.525	0.12748	0.04031	0.927 ns
		ILO	0.52	0.11353	0.0359	

## Discussion

The success of implant loading protocols is influenced by the rate of peri-implant bone loss. Therefore, the current clinical trial aimed to assess the impact of immediate and delayed implant loading protocols on peri-implant crestal bone loss in patients with implant-retained mandibular overdentures using locator attachments at two follow-up points: six and 12 months.

In this study, both immediate and delayed loading groups demonstrated a 100% implant survival rate during the twelve-month observation period. This finding aligns with previous research by Alsabeeha et al. [17], which reported a high success rate when an overdenture was supported by a single implant with locator attachments positioned in the mandibular midline utilizing a six-week loading strategy. Similarly, the delayed loading group experienced no implant failures. Another study [18] reported comparable results, where two implants were inserted in the mandible during a single surgical procedure, and mandibular overdentures were affixed to the implants using locator attachments ten weeks afterward.

The study results showed that both groups exhibited significantly greater crestal bone loss at twelve months compared to the six-month measurements. The increased bone loss over time could result from functional pressures combined with the bone's rapid reaction to healing and reorganization [19]. Among the two groups, the average crestal bone loss values at 12 months remained within the typical range documented in the literature (1 mm in the initial year) [20-22].

In the current trial, the difference in mean crestal bone loss values between the studied groups at six and 12 months was statistically insignificant. Consequently, the results support the null hypothesis of this study. Pardal-Peláez et al. [23] conducted a systematic review and meta-analysis to compare immediate versus delayed loading procedures in edentulous mandibles, examining variations in implant success rates, crestal bone loss, and evaluating outcomes based on prosthesis type and implant splinting configurations. Their meta-analysis results demonstrated an implant loss ratio of 2.63 (95% CI: 1.22, 5.68) within the first year, with delayed loading showing superior outcomes and less bone loss. They found that immediate loading procedures demonstrated a greater risk of early implant failure in comparison to delayed loading approaches. Delayed loading was favored for removable prostheses and non-splinted implants. Their review concluded that the scientific evidence strongly supports delayed loading.

Conversely, Tealdo et al. [24] examined the radiographic results and survival rates of immediate and delayed implant loading in the edentulous maxilla. In the edentulous maxilla, the immediate loading of implants inserted in both fresh extraction sockets and healed sites showed implant survival rates similar to the traditional two-stage delayed loading protocol, and even resulted in reduced crestal bone loss at three years. Suarez et al. [25] compared crestal bone loss (CBL) between implants restored using the following procedures: 1) immediate restoration/loading, 2) early loading, and 3) traditional loading. This meta-analysis indicated that implant crestal bone loss is not impacted by the restoration time and concluded that it shouldn't be the only consideration when choosing restoration treatments.

In the present investigation, the sole criterion used to assess the treatment benefits of immediate and delayed loading regimens was crestal bone loss. Nevertheless, using this sole criterion has some drawbacks. As a static metric, the raw crestal bone loss data measured after one year of loading offers only a snapshot of the peri-implant tissue condition at that specific point in time. This inherent limitation contributes to the ongoing debate regarding the validity of crestal bone loss as a sole indicator. Recognizing that crestal bone remodeling is a dynamic process, the rate of crestal bone loss has been more recently proposed as a superior index, a concept supported by research such as that conducted by Galindo-Moreno et al. [26]. The rate of crestal bone loss is measured in millimeters/month (mm/m) and may alter with time progression. According to Galindo-Moreno et al. [26], the rate of crestal bone loss tends to decrease over time. They also found that if crestal bone loss was greater than 0.44 mm at six months post-loading, the risk of implant failure was considerably elevated.

According to many studies, early exposure to oral microbiological plaque, rapid abutment attachment, and subsequent restorative movement may all compromise the early healing of implants in mandibular overdentures under an immediate loading protocol [27-29]. 

However, other researchers showed no noticeable variation in crestal bone loss surrounding immediately loaded implants in comparison to the delayed loading protocol, which is also consistent with data from other systematic reviews [30]. This finding might be explained by the early mechanical strain and the immediate loading protocol's omission of second-stage surgery [31].

This could be because mechanical stress from the overdentures in the immediate loading protocol may stimulate alveolar bone growth, leading to a higher bone-to-implant contact ratio [31]. Early mechanical strain has been found to have a positive impact on the initial stage of bone healing at the bone-implant interface [32]. Furthermore, extra stress and tissue injury from second-stage surgical procedures may result in the loss of crestal bone [33]. In comparison to an immediate loading protocol, a delayed loading protocol that requires a second-stage surgery may result in additional loss of underlying bone surrounding the implants [34].

Therefore, a comprehensive evaluation of characteristics for each implant and participant is necessary to accurately define and predict implant failure [26]. Consequently, every attempt should be made to reduce CBL surrounding implants and establish a rigorous follow-up maintenance program, considering the critical role that crestal bone loss plays in the prognosis of long-term implant success.

In a previous clinical study by Kutkut et al. [35], four locations surrounding the implants-mesial, buccal, distal, and lingual-were used to assess changes in the bone level. The study found a significant difference between the treatment groups' baseline and 12-month bone level results. Specifically, the implant-retained overdenture with immediate loading preserved more crestal bone than the implant-retained overdenture with delayed loading. This observed difference in the loss of crestal bone surrounding the implants' necks may be attributed to the bone's initial reaction to healing and reorganization over time, as well as functional stress [36]. This contrasts with our findings, as the mean values of crestal bone loss for both groups in the present study at 12 months remained within the typical range documented in the literature (1 mm in the initial year) [34, 37].

The limitations of this study include a relatively small sample size and its single-center setting, which may restrict the generalizability of the findings. Furthermore, the follow-up period was constrained to one year, potentially limiting the ability to observe long-term outcomes. Finally, a methodological consideration pertains to the radiographic measurements: all data were collected by a single calibrated examiner. Although this individual was blinded to group allocation, reliance on a single assessor precludes the calculation of inter-observer reliability (reproducibility) and prevents the independent verification of measurement accuracy. Future studies using mixed-effects models and longer observation periods are recommended.

The finding that peri-implant crestal bone loss with Immediate Loading (IL) protocols is statistically equivalent to that observed with Delayed Loading (DL) protocols over a one-year follow-up period constitutes a fundamental validation of the IL approach. This outcome effectively mitigates the primary biological concern historically associated with immediate functional stress on newly placed mandibular implants.

The key clinical implication is that the IL protocol, provided strict adherence to primary stability prerequisites is maintained, can be confidently adopted as the preferred treatment strategy for the mandibular overdenture. This strategic shift allows clinicians to simultaneously achieve the high biological success rates expected of implant therapy while providing patients with immediate functional benefits and maximizing their quality of life.

## Conclusions

Within the limitations of this study, it can be concluded that the peri-implant crestal bone loss associated with the immediate loading protocol for implant-retained mandibular overdentures utilizing locator attachments demonstrated no statistically significant difference when compared to the delayed loading protocol at the one-year follow-up period. This suggests that immediate loading can be a viable alternative to delayed loading in clinical practice, offering benefits such as reduced treatment time and enhanced patient satisfaction without compromising peri-implant bone levels.
